# Reevaluating the prognostic significance of male gender for papillary thyroid carcinoma and microcarcinoma: a SEER database analysis

**DOI:** 10.1038/s41598-017-11788-8

**Published:** 2017-09-12

**Authors:** Chunping Liu, Tianwen Chen, Wen Zeng, Shuntao Wang, Yiquan Xiong, Zeming Liu, Tao Huang

**Affiliations:** 1 Department of Breast and Thyroid Surgery, Union Hospital, Tongji Medical College, Huazhong University of Science and Technology, Wuhan 430022, People’s Republic of China; 2 Department of Breast and Thyroid Surgery, Affiliated Nanshan Hospital, Guangdong Medical University, Shenzhen, China; 3 Department of Ophthalmology, Zhongnan Hospital, Wuhan University, Wuhan, Hubei China

## Abstract

The prognostic significance of gender remains controversial for papillary thyroid carcinoma (PTC). In this study, we investigated the associations between gender and prognosis in a large cohort of patients with PTC or PTMC that was diagnosed in 2010–2013 and recorded in the Surveillance, Epidemiology, and End Results cancer registry. The mean ± standard deviation duration of survival for all patients with PTC during the study period was 21.47 ± 14.04 months. In Kaplan-Meier analyses of the entire cohort of PTC patients, survival curves for all-cause death and cancer-specific death declined more sharply for men than for women. Similar results were observed in analyses of patients with PTCs > 1 cm and PTMC. After adjusting for potential confounders, hazard rates indicated significantly elevated all-cause mortality for men in analyses of all PTCs, PTCs > 1 cm, and PTMCs. However, in a confounder-adjusted analysis of patients with PTMC, the hazard rate did not indicate significantly higher mortality for men than for women. Our study demonstrated that male gender is an independent poor prognostic factor for all PTCs and for PTCs > 1 cm. However, gender is not an independent prognostic factor for cause-specific survival in PTMC.

## Introduction

The incidence rate of thyroid cancer rose rapidly in recent decades^1^. However, the incidence rate of thyroid cancer also appears to have begun to stabilize in recent years, since changes in clinical practice guidelines were initiated in 2009^2^. Papillary thyroid cancer (PTC) accounts for 80–90% of all thyroid malignancies, making it the most common type of thyroid malignancy^3–5^.

According to World Health Organization guidelines, papillary thyroid microcarcinoma (PTMC) is defined as thyroid carcinoma measuring less than or equal to 1 cm in its greatest dimension^6^. In recent reports, roughly 18.4% to 50% of the increase in PTC has been attributed to the identification of intrathyroidal papillary thyroid microcarcinomas^7–11^.

According to previous studies, it is still controversial whether male gender a poor prognostic factor in cases of PTC^12, 13^. A recent meta-analysis demonstrated that male gender was a strong prognostic factor, and increased the risk of recurrence^14^. Because of the contradictory results in the prior literature, a large-scale population-based cohort should be investigated to clarify the associations between gender and the prognoses of PTC and PTMC.

The Surveillance, Epidemiology, and End Results (SEER) program of the National Cancer Institute (NCI) is the largest publicly available and authoritative source of data on cancer incidence and survival. In the present study, we used this reliable and large-scale research dataset to reevaluate the prognostic significance of male gender for PTC and PTMC. Our analysis was based on the records of 43712 patients with PTCs diagnosed during 2010–2013.

## Results

### Demographic and clinical features

Baseline clinicopathologic characteristics are summarized and compared between men and women with PTC in Table 1. The mean ± standard deviation (SD) durations of survival during the study period was 21.47 ± 14.04 months for the entire cohort of patients with PTC. Mean age was higher (53.03 ± 15.30 vs 48.68 ± 15.14 years, *p* < 0.001) and tumor size was larger (22.48 ± 21.79 vs 16.93 ± 17.75 mm, *p* < 0.001) in men than in women. In patients with PTC, gender was associated with race, T stage, N stage, distant metastasis, extrathyroidal extension, multifocality, radiation, and surgical method (Table 1).

**Table 1:** Characteristics of Patients with Papillary Thyroid Cancer from the Surveillance, Epidemiology, and End Results Database (2010–2013).

Characteristic	Variable	Female (n = 33614)	Male (n = 10098)	*P*
Age	Mean ± SD	48.68 ± 15.14	53.03 ± 15.30	<0.001
	<45 year	13597(40.5%)	2962(29.3%)	<0.001
	≥ 45 year	20017(59.5%)	7136(70.7%)	
Race	white	26843(81.1%)	8484(85.1%)	<0.001
	black	2381(7.2%)	502(5.0%)	
	other	3875(11.7%)	989(9.9%)	
Tumor size	Mean ± SD	16.93 ± 17.75	22.48 ± 21.79	<0.001
	≤ 1.0 cm	14425(42.9%)	3347(33.1%)	<0.001
	> 1.0 cm	19189(57.1%)	6751(66.9%)	
T stage	T1	21435(64.0%)	5203(51.7%)	<0.001
	T2	5293(15.8%)	1794(17.8%)	
	T3	5956(17.7%)	2595(25.8%)	
	T4	826(2.5%)	473(4.7%)	
N stage	N0	26685(80.7%)	7047(70.9%)	<0.001
	N1	6391(19.3%)	2889(29.1%)	
Distant metastasis	No	33347(99.2%)	9861(97.7%)	<0.001
	Yes	267(0.8%)	236(2.3%)	
Extrathyroidal extension	No	26225(84.9%)	7263(79.1%)	<0.001
	Yes	4664(15.1%)	1915(20.9%)	
Multifocal tumor	No	19884(59.8%)	5525(55.5%)	<0.001
	Yes	13355(40.2%)	4436(44.5%)	
Surgery	No^a^	427(1.3%)	201(2.0%)	<0.001
	Yes^b^	33107(98.7%)	9827(98.0%)	
Radiation	None or refused	17793(54.3%)	4689(47.8%)	<0.001
	performed	14958(45.7%)	5111(52.2%)	
All cause death	No	33128(98.6%)	9706(96.1%)	<0.001
	Yes	486(1.4%)	392(3.9%)	
Cancer-specific death	No	33497(99.7%)	9991(98.9%)	<0.001
	Yes	117(0.3%)	107(1.1%)	

^a^Recommended but not Performed OR Not recommended; ^b^Performed.

Baseline clinicopathologic characteristics are summarized and compared between men and women with PTC > 1 cm in Table 2. The mean ± SD duration of survival for patients with PTC > 1 cm was 21.52 ± 14.00 months. Mean age was higher (52.26 ± 15.98 vs 47.31 ± 15.83 years, *p* < 0.001) and tumor size was larger (25.53 ± 19.32 vs 30.90 ± 22.18 mm, *p* < 0.001) in men than in women. PTC > 1 cm in male patients was more frequently associated with race, T stage, N stage, distant metastasis, extrathyroidal extension, multifocality, radiation, and surgical method (Table 2).

**Table 2:** Characteristics of Patients with Papillary Thyroid Cancer ≥ 1 cm from the Surveillance, Epidemiology, and End Results Database (2010–2013).

Characteristic	Variable	Female (n = 19189)	Male (n = 6751)	*P*
Age	Mean ± SD	47.31 ± 15.83	52.26 ± 15.98	<0.001
	<45 year	8653(45.1%)	2178(32.3%)	<0.001
	≥45 year	10536(54.9%)	4573(67.7%)	
Race	white	15098(79.9%)	5648(84.7%)	<0.001
	black	1347(7.1%)	327(4.9%)	
	other	2452(13.0%)	694(10.4%)	
Tumor size	Mean ± SD	25.53 ± 19.32	30.90 ± 22.18	<0.001
T stage	T1	7891(41.3%)	2105(31.3%)	<0.001
	T2	5293(27.7%)	1794(26.7%)	
	T3	5155(27.0%)	2369(35.3%)	
	T4	759(4.0%)	452(6.7%)	
N stage	N0	13727(73.2%)	4300(65.1%)	<0.001
	N1	5031(26.8%)	2309(34.9%)	
Distant metastasis	No	18945(98.7%)	6539(96.9%)	<0.001
	Yes	244(1.3%)	211(3.1%)	
Extrathyroidal extension	No	13400(77.6%)	4323(71.9%)	<0.001
	Yes	3877(22.4%)	1690(28.1%)	
Multifocal tumor	No	10560(55.9%)	3503(52.7%)	<0.001
	Yes	8355(44.1%)	3139(47.3%)	
Surgery	No^a^	335(1.8%)	173(2.6%)	<0.001
	Yes^b^	18799(98.2%)	6548(97.4%)	
Radiation	None or refused	7045(37.8%)	2309(35.3%)	<0.001
	performed	11570(62.2%)	4228(64.7%)	
All cause death	No	18851(98.2%)	6479(96.0%)	<0.001
	Yes	338(1.8%)	272(4.0%)	
Cancer-specific death	No	19080(99.4%)	6651(98.5%)	<0.001
	Yes	109(0.6%)	100(1.5%)	

^a^Recommended but not Performed OR Not recommended; ^b^Performed.

Next, we focused on the baseline clinicopathologic characteristics of patients with PTMC, which are summarized in Table 3. The mean ± SD survival months for PTMC patients was 21.38 ± 14.09 months. Mean age was higher (50.50 ± 13.96 vs 54.61 ± 13.71 years, *p* < 0.001) and tumor size was larger (5.49 ± 2.94 vs 5.49 ± 2.98 mm, *p* = 0.0909) in men than in women. PTMC in male patients was more frequently associated with race, T stage, N stage, distant metastasis, extrathyroidal extension, multifocality, and radiation (all p < 0.05), but not with surgical method (p = 0.189) (Table 3).

**Table 3:** Characteristics of Patients with Papillary Thyroid Microcarcinoma from the Surveillance, Epidemiology, and End Results Database (2010–2013).

Characteristic	Variable	Female (n = 14425)	Male (n = 3347)	*P*
Age	Mean ± SD	50.50 ± 13.96	54.61 ± 13.71	<0.001
	<45 year	4944(34.3%)	784(23.4%)	<0.001
	≥ 45 year	9481(65.7%)	2563(76.6%)	
Race	white	11745(82.7%)	2836(85.8%)	<0.001
	black	1034(7.3%)	175(5.3%)	
	other	1423(10.0%)	295(8.9%)	
Tumor size	Mean ± SD	5.49 ± 2.94	5.49 ± 2.98	0.909
T stage	T1	13544(94.0%)	3098(92.6%)	0.013
	T3	801(5.6%)	226(6.8%)	
	T4	67(0.4%)	21(0.6%)	
N stage	N0	12958(90.5%)	2747(82.6%)	<0.001
	N1	1360(9.5%)	580(17.4%)	
Distant metastasis	No	14402(99.8%)	3322(99.3%)	<0.001
	Yes	23(0.2%)	25(0.7%)	
Extrathyroidal extension	No	12825(94.2%)	2940(92.9%)	0.005
	Yes	787(5.8%)	225(7.1%)	
Multifocal tumor	No	9324(65.1%)	2022(60.9%)	<0.001
	Yes	5000(34.9%)	1297(39.1%)	
Surgery	No^a^	92(0.6%)	28(0.8%)	0.189
	Yes^b^	14308(99.4%)	3279(99.2%)	
Radiation	None or refused	10748(76.0%)	2380(72.9%)	<0.001
	performed	3388(24.0%)	883(27.1%)	
All cause death	No	14277(%)	3227(%)	<0.001
	Yes	148(%)	120(%)	
Cancer-specific death	No	14417(99.9%)	3340(99.8%)	0.006
	Yes	8(0.1%)	7(0.2%)	

^a^Recommended but not Performed OR Not recommended; ^b^Performed.

### Survival analysis of patients with PTC

**Figure 1:**
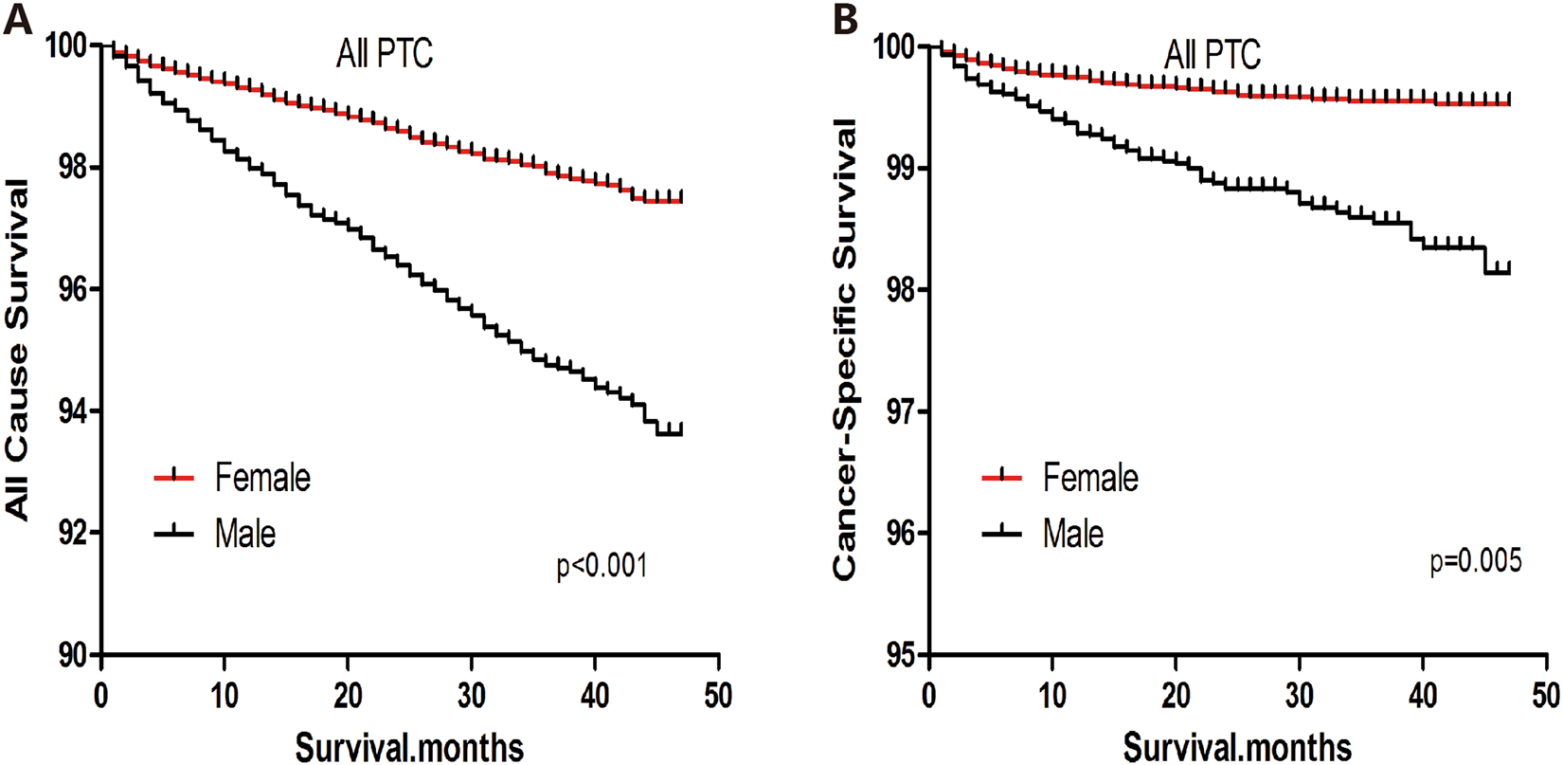
All-cause survival (**A**) and cancer-specific survival (**B**) for men and women with papillary thyroid carcinoma.

**Figure 2:**
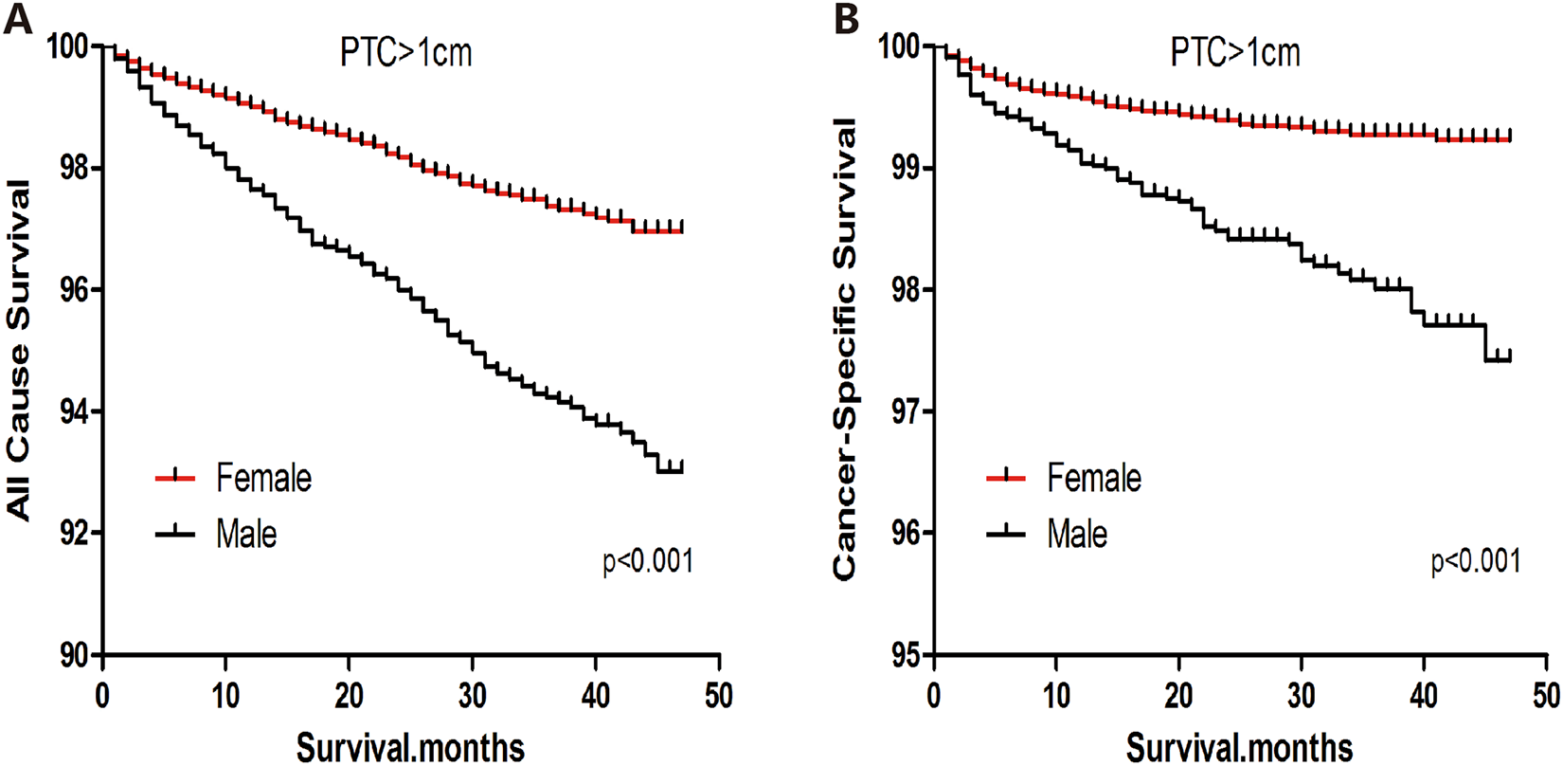
All-cause survival (**A**) and cancer-specific survival (**B**) for men and women with papillary thyroid carcinomas >1 cm.

**Figure 3:**
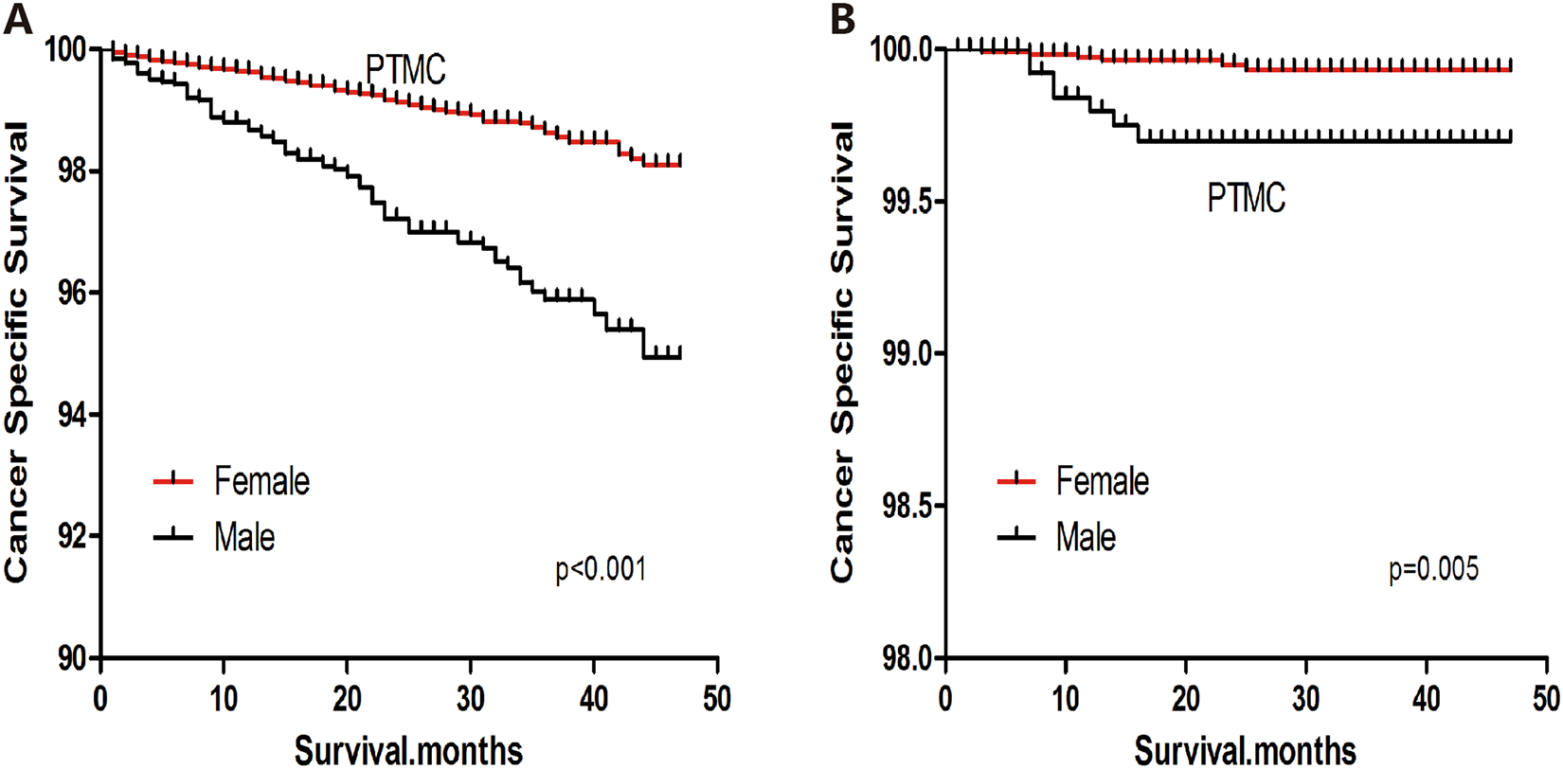
All-cause survival (**A**) and cancer-specific survival (**B**) for men and women with papillary thyroid microcarcinoma.

In a Kaplan-Meier analysis of the entire cohort of PTC patients, all-cause survival declined sharply for men, but declined more modestly for women (Log-rank test, p < 0.001) (Fig. 1A). Similar results were observed for cause-specific survival (Log-rank test, p = 0.005) (Fig. 1B). Among patients with PTC > 1 cm, all-cause and cause-specific survival curves were poorer for men than women (Log-rank test, both p < 0.001) (Fig. 2A,B). Among patients with PTMC, all-cause and cause-specific survival curves were also poorer for men than women (Log-rank test; all-cause death, p < 0.001; cancer-specific death, p = 0.005) (Fig. 3A,B).

In the entire cohort of patients with PTC, the rate of all-cause death per 1000 person-years was 7.16 (95% confidence interval [CI], 6.52-7.87) for men and 18.28 (95% CI, 16.40-20.37) for women (Table 4). Comparing male vs. female patients, the hazard ratio (HR) for all-cause mortality was 2.53 (95% CI, 2.20-2.90). After adjustment for patient age at diagnosis, race, multifocality, tumor size, extrathyroidal invasion, lymph node metastasis, distant metastasis, radiation, and surgical treatment, these HRs for all-cause and cause-specific mortality became 1.63 (95% CI, 1.39-1.91) and 1.67 (95% CI, 1.42-1.97), respectively. Therefore, male gender had a robust effect on all-cause death in the overall cohort of PTC patients; this effect remained even after additional adjustment for conventional clinicopathological risk factors and treatment methods (Table 4). We subsequently investigated all of the abovementioned associations in subgroup analyses of patients with PTC > 1 cm and PTMC. Similar results were observed (Table 4).

**Table 4:** Hazard Ratios of sex for the all cause deaths of PTC.

Versions	Sex	All Cause Deaths, No.	%	All Cause Deaths per 1,000 Person-Years	95% CI	Unadjusted			Adjustment 1*			Adjustment 2^†^		
						Hazard Ratios	95% CI		Hazard Ratios	95% CI		Hazard Ratios	95% CI	
All PTC	Female	486	1.45	7.16	6.52–7.87	1 [Reference]			1 [Reference]			1 [Reference]		
	Male	392	3.89	18.28	16.40–20.37	2.53	2.20–2.90	<0.001	1.63	1.39–1.91	<0.001	1.67	1.42–1.97	<0.001
PTC > 1 cm	Female	338	1.76	9.13	8.17–10.20	1 [Reference]			1 [Reference]			1 [Reference]		
	Male	272	4.03	20.56	18.16–23.28	2.27	1.93–2.66	<0.001	1.46	1.21–1.77	<0.001	1.52	1.25–1.84	<0.001
PTMC	Female	148	1.03	4.54	3.78–5.44	1 [Reference]			1 [Reference]			1 [Reference]		
	Male	120	3.59	13.50	10.81–16.85	2.84	2.16–3.73	<0.001	2.07	1.54–2.78	<0.001	2.06	1.52–2.77	<0.001

PTC: Papillary thyroid cancer; PTMC: Papillary Thyroid Microcarcinoma.

*Adjustment 1 was made for patient age at diagnosis, race, multifocality, tumor size, extrathyroidal invasion, lymph node metastasis and distant metastasis.

^†^Adjustment 2 was made for patient age at diagnosis, race, multifocality, tumor size, extrathyroidal invasion, lymph node metastasis, distant metastasis, radiation and surgery treatment.

Furthermore, we analyzed cancer-specific deaths. In the entire cohort of patients with PTC, the rate of cancer-specific deaths per 1000 person-years was 1.76 (95% CI, 1.46-2.13) for men and 5.42 (95% CI, 4.44-6.62) for women (Table 5). Comparing male vs. female patients, the HR for cancer-specific deaths was 3.09 (95% CI, 2.38-4.02). After adjustment for conventional clinicopathological risk factors and treatment methods, the HRs for cancer-specific deaths were 1.50 (95% CI, 1.10-2.06) and 1.53 (95% CI, 1.11-2.10), respectively. Similar results were observed for patients with PTC > 1 cm. However, among patients with PTMC, the HR for cancer-specific deaths was 3.89 (95% CI, 1.41-10.71) without adjustment and 1.23 (95% CI, 0.36-4.16; p = 0.743) after adjustment for patient age at diagnosis, race, multifocality, tumor size, extrathyroidal invasion, lymph node metastasis, and distant metastasis. Moreover, after the HR became 0.77 (95% CI, 0.19-3.16; p = 0.769) after adjustment for patient age at diagnosis, race, multifocality, tumor size, extrathyroidal invasion, lymph node metastasis, distant metastasis, radiation, and surgical treatment (Table 5).

**Table 5:** Hazard Ratios of sex for the cancer specific deaths of PTC.

Versions	Sex	Cancer Specific Deaths, No.	%	Cancer Specific Deaths per 1,000 Person–Years	95% CI	Unadjusted			Adjustment 1*			Adjustment 2^†^		
						Hazard Ratios	95% CI	*p*	Hazard Ratios	95% CI	*p*	Hazard Ratios	95% CI	*p*
All PTC	Female	117	0.35	1.76	1.46–2. 13	1 [Reference]			1 [Reference]			1 [Reference]		
	Male	107	1.06	5.42	4.44–6.62	3.09	2.38–4.02	<0.001	1.50	1.10–2.06	0.011	1.53	1.11–2.10	0.010
PTC > 1 cm	Female	109	0.57	2.91	2.39–3.54	1 [Reference]			1 [Reference]			1 [Reference]		
	Male	100	1.48	7.43	6.04–9.14	2.62	2.00–3.45	<0.001	1.46	1.05–2.02	0.024	1.52	1.09–2.11	0.014
PTMC	Female	8	0.06	0.23	0.10–0.52	1 [Reference]			1 [Reference]			1 [Reference]		
	Male	7	0.21	1.21	0.58–2.54	3.89	1.41–10.71	0.009	1.23	0.36–4.16	0.743	0.77	0.19–3.16	0.769

PTC: Papillary thyroid cancer; PTMC: Papillary Thyroid Microcarcinoma.

*Adjustment 1 was made for patient age at diagnosis, race, multifocality, tumor size, extrathyroidal invasion, lymph node metastasis and distant metastasis.

^†^Adjustment 2 was made for patient age at diagnosis, race, multifocality, tumor size, extrathyroidal invasion, lymph node metastasis, distant metastasis, radiation and surgery treatment.

## Discussion

Several studies have reported conflicting results regarding the effects of male gender on the prognosis of PTC. Kruijff and colleagues demonstrated the risk of structural recurrence in men was 2.44 times that in women, with a median follow-up period of 31 months^15^. A meta-analysis also showed that male gender was an independent poor prognostic factor, and that the risk of recurrence in men was 1.53 times that in women^14^. However, only 4 of the 13 articles included in this meta-analysis reported that male gender was a poor prognostic factor for PTC.

Oyer *et al*. concluded that there was no difference in disease-specific survival between male and female patients with the same disease stage, according to an analysis of the SEER database from 1988–2003^16^. Nilubol and Grogan *et al*. also reported that male gender was not an independent prognostic factor of cancer specific survival in PTC patients^12, 17^. Similar result was also reported by Matsuzu *et al*.’s study which recorded 1088 PTC patients with a median follow-up period of 17.6 years^18^.

These inconsistent results introduce a dilemma into the treatment of PTC. More aggressive treatment options (such as total thyroidectomy, or lymph node dissection followed by radioactive iodine ablation) should be considered for men if male gender is a negative independent prognostic factor in PTC. The purpose of this study was to investigate whether male gender was a poor prognostic factor in PTC. Throughout our analyses of the SEER database, male gender was associated with elevated risks of all-cause death and cancer-specific death, both for PTC as a whole and for PTCs > 1 cm. Therefore, based on our results, relatively more aggressive treatment should be considered for these subgroups of patients.

Among patients with PTMC, male gender was associated with significantly elevated risks of all-cause death. However, we found that this association did not remain statistically significant after adjusting for patient age at diagnosis, race, multifocality, and other risk-associated clinicopathological characteristics and treatment methods. This may be attributable to the extremely good prognosis of PTMC, which as 15-year disease-specific survival rates as high as 99% and overall survival of 97.5%^19, 20^.

Recent studies have reported that estrogen can regulate thyroid cell proliferation by binding to both ERα, and ERβ. These studies attempted to investigate the associations between estrogen receptor (ER) expression and tumor aggressiveness and prognosis in PTC, but obtained inconsistent conclusions^21–23^. Yi *et al*. investigated the clinical implications of ER coding genes in PTC and found that ESR1 expression and the ESR ratio were associated with aggressive clinicopathological factors and poor overall survival in women^21^. Huang *et al*. further observed that ERα stimulates PTC growth and progression, whereas ERb1 inhibits PTC growth and progression^23^. Since ER expression levels differ between men and women, we speculate that ER level may be one of the underlying reasons for the gender differences in PTC tumor aggressiveness and prognosis. However, further research (including both genomic and epidemiologic investigations) should be performed to reveal the source of the gender disparity.

Some inherent limitations must be taken into consideration in the interpretation of our results. The major limitations of this study are that data regarding recurrence are not captured in SEER, and that designation of cancer-specific death is susceptible to overestimation bias, particularly for diseases such as PTMC. Furthermore, vascular invasion, family history, and other histologic findings were not evaluated or included in our study. In addition, the molecular markers such as *BRAF* point mutation and *TERT* promotor point mutation, were not obtained in our study or adjusted for in our analyses. Furthermore, given the generally favorable prognosis of PTC, the relatively short study period and follow-up (2010–2013) is a limitation to our analysis.

## Conclusion

Our study demonstrated that male gender is an independent poor prognostic factor for all PTCs and for PTCs > 1 cm. However, gender could actually have substantial prognostic relevance for PTMC, but that you do not have enough data or a long enough follow-up period to determine this reliably. These results may be instructive for clinicians. In general, more aggressive treatments should be provided to men with all PTCs and PTCs > 1 cm.

## Methods and Materials

### Database

We investigated PTC and PTMC in a large cohort of patients from SEER. The SEER project is a United States population-based cancer registry that began in 1973 and is supported by the National Cancer Institute and the Centers for Disease Control and Prevention. It covers approximately 30% of the population of the United States and contains data across multiple geographic regions on incidence, prevalence, mortality, population-based variables, primary characteristics of the tumor, and other attributes.

### Data collection and analysis

We examined SEER data for 2010–2013 and selected patients with a diagnosis of PTC, as defined by a combination of ICD-O site code of C73.9 (i.e., thyroid) and papillary histology. The following diagnostic codes were included in the study: “papillary carcinoma,” “papillary adenocarcinoma,” “oxyphilic adenocarcinoma”, “papillary adenocarcinoma,” and “papillary cyst-adenocarcinoma.” Demographic information, age, sex, tumor size, extrathyroidal extension, multifocality, nodal, metastasis, surgical treatment and radiation treatment were compiled from the SEER dataset, and a survival analysis was performed to evaluate the associations between gender and prognosis.

### Statistical analysis

Patients were followed up until December 2013. The outcomes measures were thyroid carcinoma-specific mortality and all-cause mortality. Patient survival curves were investigated using Kaplan-Meier analyses, log-rank tests, and Cox proportional hazards regression analyses. HRs were used to show the magnitude of the effect of stages on cancer-specific mortality. Ninety-five percent CIs were used to indicate the significance of the risks. All P values were 2-sided and P less than .05 was regarded as indicating statistical significance. Analyses were performed using SPSS version 19.0 (IBM Corp, Armonk, NY, USA), Stata/SE version 12 (Stata Corp, College Station, TX, USA), and GraphPad Prism version 6 (GraphPad Software Inc, La Jolla, CA, USA).
